# Excitement and Concerns of Young Radiation Oncologists over Automatic Segmentation: A French Perspective

**DOI:** 10.3390/cancers15072040

**Published:** 2023-03-29

**Authors:** Vincent Bourbonne, Adrien Laville, Nicolas Wagneur, Youssef Ghannam, Audrey Larnaudie

**Affiliations:** 1 Radiation Oncology Department, University Hospital Brest, 2 Avenue Foch, 29200 Brest, France; 2 Société Française des Jeunes Radiothérapeutes Oncologues, 47 Rue de la Colonie, 75013 Paris, France; 3 Radiation Oncology Department, University Hospital Amiens-Picardie, 30 Avenue de la Croix Jourdain, 80054 Amiens, France; 4 Radiation Oncology Department, Institut de Cancérologie de l’Ouest, Centre Paul Papin, 15 Rue André Bocquel, 49055 Angers, France; 5 Radiation Oncology Department, Centre François Baclesse, 3 Avenue du Général Harris, 14000 Caen, France

**Keywords:** artificial intelligence, automatic segmentation, radiation oncology

## Abstract

**Simple Summary:**

Radiation oncology has known tremendous technological advances in recent years, the latest being brought by artificial intelligence (AI). Applied to segmentation, some concerns were raised by academics regarding the impact on young radiation oncologists’ training. To answer those concerns, a survey was conducted by the SFjRO (Société Française des jeunes Radiothérapeutes Oncologues). The survey was mandatory for registration to a dosimetry webinar dedicated to head and neck cancers. A significant time gain was observed for the delineation of organs at risk, with almost 35% of the participants saving between 50–100% of the segmentation time, while only 8.6% experienced such a gain for the delineation of target volumes. The majority of participants suggested that these tools should be integrated into the training so that future radiation oncologists do not neglect the importance of radioanatomy. Fully aware of this risk, up to one-third of them even suggested that AI tools should be reserved for senior physicians only.

**Abstract:**

Introduction: Segmentation of organs at risk (OARs) and target volumes need time and precision but are highly repetitive tasks. Radiation oncology has known tremendous technological advances in recent years, the latest being brought by artificial intelligence (AI). Despite the advantages brought by AI for segmentation, some concerns were raised by academics regarding the impact on young radiation oncologists’ training. A survey was thus conducted on young french radiation oncologists (ROs) by the SFjRO (Société Française des jeunes Radiothérapeutes Oncologues). Methodology: The SFjRO organizes regular webinars focusing on anatomical localization, discussing either segmentation or dosimetry. Completion of the survey was mandatory for registration to a dosimetry webinar dedicated to head and neck (H & N) cancers. The survey was generated in accordance with the CHERRIES guidelines. Quantitative data (e.g., time savings and correction needs) were not measured but determined among the propositions. Results: 117 young ROs from 35 different and mostly academic centers participated. Most centers were either already equipped with such solutions or planning to be equipped in the next two years. AI segmentation software was mostly useful for H & N cases. While for the definition of OARs, participants experienced a significant time gain using AI-proposed delineations, with almost 35% of the participants saving between 50–100% of the segmentation time, time gained for target volumes was significantly lower, with only 8.6% experiencing a 50–100% gain. Contours still needed to be thoroughly checked, especially target volumes for some, and edited. The majority of participants suggested that these tools should be integrated into the training so that future radiation oncologists do not neglect the importance of radioanatomy. Fully aware of this risk, up to one-third of them even suggested that AI tools should be reserved for senior physicians only. Conclusions: We believe this survey on automatic segmentation to be the first to focus on the perception of young radiation oncologists. Software developers should focus on enhancing the quality of proposed segmentations, while young radiation oncologists should become more acquainted with these tools.

## 1. Introduction

Radiation oncology has known tremendous technological advances in recent years [1,2,3], the latest being brought by artificial intelligence (AI) [4,5,6,7]. AI is taking more and more place in the clinical workflow of patients undergoing radiotherapy (RT), from segmentation to dosimetry planning and prediction modeling of clinical endpoints. Segmentation is a major and time-consuming step, mixing repetitive steps, such as the delineation of organs at risk (OARs), but also experience needing tasks for the delineation of target volumes. Automatic segmentation can use atlas-based methods with single-atlas, multi-atlas, or a hybrid. Deep learning techniques have been recently applied [8]. A strong focus was made on head and neck (H & N) delineation due to its particular long time of contouring, estimated at around 2.7–3 h without AI help [9]. Although the delineation of OARs requires anatomical knowledge, there is a little inter-individual variation in their definition. However, when defining the target volumes (gross tumor volume and clinical target volume), one must adapt to the patient’s anatomy as well as the disease characteristics, often using multimodal information and imaging. The lack of reliable and robust data is the main limitation of such tasks.

Some concerns were raised by academics regarding the consequences of young radiation oncologists’ training. The impact of automated segmentation tools depends on the contours. For OARs, in addition to the loss of technical skills and dependence on computer tools, the extensive use of automatic segmentation tools could lead to a lack of knowledge of radioanatomy and, therefore, an inability to correct the proposed segmentations resulting in a blind trust. For target volumes, delineation takes into account the international guidelines but also the radiation oncologist’s experience and all the patient’s characteristics, data that are difficult to learn for an AI tool.

The Société Française des jeunes Radiothérapeutes Oncologues (SFjRO) is a national association reuniting all the residents and young radiation oncologists (ROs) funded in 2002 [10]. Its main goals include radiation oncology (RO) training and working in close collaboration with the french society of Radiation Oncology (SFRO) and the National Board of Oncology Teachers (CNEC). Translational lectures are given several times a year to improve the training of RO residents [11,12,13,14]. Concerning delineation topics, national courses are organized regularly in radio-anatomy with around 180–200 attendings (Paris 2009, Paris 2012, Paris 2018). Due to the importance and evolution of contouring guidelines, webinars and online delineation courses are proposed.

Given the broader use of AI segmentation, the radiation oncologist’s job and his/her daily tasks are rapidly evolving. As there were only scarce data in the literature concerning young ROs, a survey was thus conducted by the SFjRO. After giving an overview of the participants and the use of segmentation AI tools in France, this article consists of the presentation of the main results, projecting on a possible better integration of AI in radiation oncology training.

## 2. Methodology—Form

The SFjRO organizes regular webinars focusing on anatomical localization, discussing either segmentation or dosimetry in collaboration with Dline.io (https://dline.io). In November 2022, the SFjRO conducted a dosimetry webinar addressing H & N cancers. A Google form was distributed and mandatory for registration to the webinar. It consisted of 22 questions formulated in French as presented in Supplementary Protocol S1. The SFjRO board is composed of 15 young radiation oncologists with different levels of experience. As a member of the board, Vincent Bourbonne was in charge of the questionnaire. Before choosing the questions, several goals were defined:

- Give an overview of the use of automatic segmentation tools in France;
- Give an overview of the pros and cons of such tools;
- Give insight into the future developments of AI tools for radiation oncologists.

Vincent Bourbonne and a second radiation oncologist (Adrien Laville) designed the questionnaire. Before being released, the questionnaire was internally validated by all co-authors but also the full SFjRO board. All members of the board took the test before its release to assess its readability and consistency. The validation process took three months, from the initial conception of the questionnaire to the final validation. The survey was compliant with the CHERRIES guidelines [15] as detailed in Supplementary Table S1. For completeness, the English translation is hereby presented in Table 1. Quantitative data (e.g., time savings and correction needs) were not measured but determined among the propositions.

## 3. Results

### 3.1. Participants

A total number of 117 participants registered for the dosimetry webinar, with a high majority of residents (99/117, 84.6%) followed by attendings (11/117, 9.4%). The rest were relatively well distributed between a single student, senior physicians (2/117, 1.7%) and foreign students (3/117, 2.7%). The majority of residents were 3rd-, 4th- or 5th-year residents (28.3%, 24.2% and 30.3%, respectively). First- and second-year residents accounted for 17.1% of the overall participants. All of the eleven attendings had less than 2 years of experience while 85.7% of the senior physicians (6/7) had 2 to 5 years of experience.

The very high majority of participants exercised in academic centers, with 48.7% (57/117) and 47% (55/117) being employed by a university hospital or an academic cancer care facility, respectively. These 35 centers were high-volume centers (on a French base) considering the number of new patients treated per year, with 35.9% of the participants working in a center with >2000 patients per year and 27.4% in centers with 1500–2000 patients per year (Supplementary Figure S1).

For 76.1% of the participants, OARs were mostly delineated by residents, while 60.7% of the centers were equipped with an automatic segmentation solution (71/117). For this subset of participants, the acquisition of the solution was fairly recent, with 40.8% and 33.8% of the participants having less than one year or 1 to 2 years of experience with AI solutions, respectively. Only 25.4% (18/71) were equipped with automatic segmentation software for more than 2 years. The large majority of participants declared that their center was planning to be equipped with such solutions (91.3%), with 52.2% of all participants to be equipped in the next 2 coming years.

Access to the AI software was non-restricted for 45.1% of the participants, with physicians, residents, physicists, and dosimetrists having access to the solution. For 49.3% of the participants, the solution was only available for residents and physicians, while only 4.2% had restricted access for senior physicians only (Supplementary Figure S2).

### 3.2. Use of Automatic Segmentation in France

#### 3.2.1. Overview of AI-Based Segmentation Solutions Used in France

**Figure 1:**
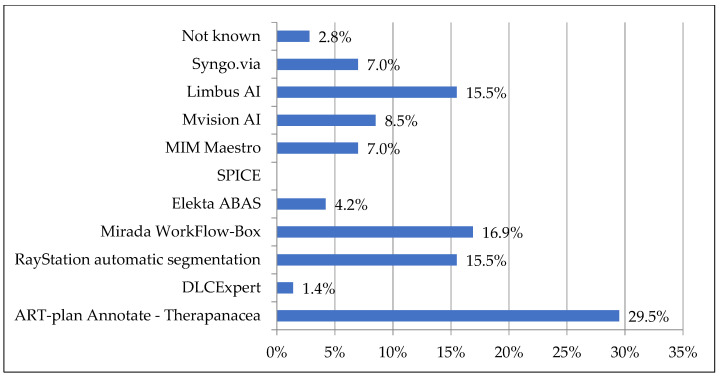
Distribution of automatic segmentation solutions in France, on a participant-by-participant analysis.

The solution provided by Therapanacea appeared as the most used software (29.5%), followed by Mirada Workflow-Box (16.9%), Raystation automatic segmentation (15.5%) and Limbus AI (15.5%), as presented in Figure 1. Of note, several centers had multiple solutions.

Seventy participants (98.6%) declared that time-saving was a motivation for the acquisition of such tools, while 35.2% saw automatic segmentation as a tool to reduce inter-physician variability. Enhancing the quality of treatment planning and leaning towards international guidelines were reasons for only 15.5% and 16.9% of the participants, respectively.

#### 3.2.2. Clinical Use of AI-Based Segmentation Tools

The localizations in which AI tools were mostly used were breast (57.7%) and head and neck (57.7%), closely followed by prostate (49.3%) and thorax (46.5%). Neurological and abdominal localizations were the two indications in which the AI tools were used less (35.2% and 26.8%). Automatic segmentation was also rarely used in palliative settings (19.7%).

**Figure 2:**
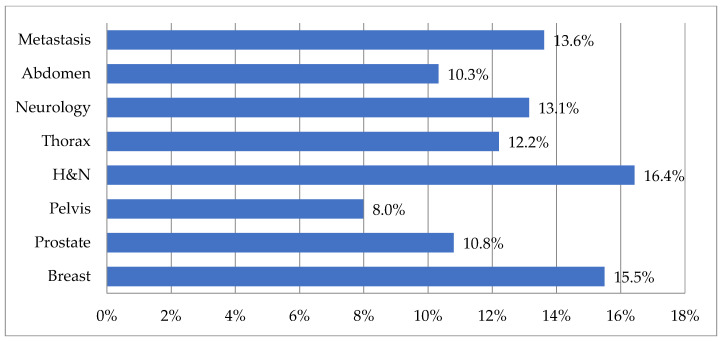
Ranking of localizations based on their time-saving potential—Focus on 6–8 rankings.

Regarding the time saved on a localization-by-localization approach, conflicting results were observed. Breast and metastatic sites were classified as the least time-saving localizations for 22.5% and 28.2% of the participants but also classified as the most time-saving localizations for 23.9% and 28.2% of the participants. When focusing on the moderate to most time-saving localizations (ranked as 6 to 8 on a 1 to 8 scale), H & N appeared as the most time-saving localization (16.4%), followed by breast (15.5%), as seen in Figure 2.

**Figure 3:**
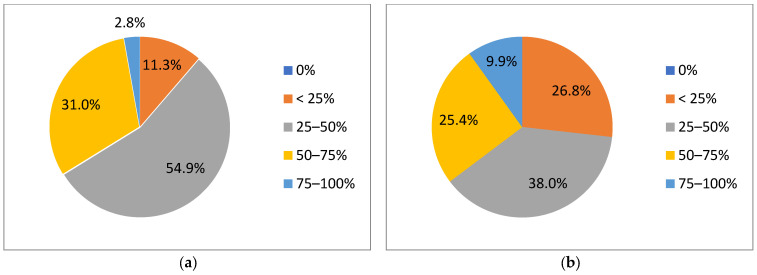
Estimation of time savings (**a**) and correction times (**b**) regarding organs at risk segmentation.

Time-saving occurred on the delineation of OARs for 93% of the participants, largely higher than prophylactic lymph node delineation (23.9%). Time gained using AI segmentation tools was estimated to be between 25–50% for 54.9% of the participants, while 31% of them even described a 50–75% time saving, correction included (Figure 3a). The time savings could probably be enhanced given the fact that 38% and 25.4% of the participants, respectively, estimated that 25–50% to 50–75% of the slices needed editing (Figure 3b). Only 26.8% of the participants answered that less than 25% of the slices needed correction.

The vast majority of participants noticed small and easily corrected mistakes (62%). Noticeably, up to 11.3% and 40.8% of the participants, respectively, reported laterality errors or aberrant segmentations such as wrong localizations.

### 3.3. Definition of Organs at Risk

Participants were asked to choose three OARs from a proposed list for each localization (brain, H & N, thorax, abdomen and pelvis). The overall list of OARs is detailed in the survey. Regarding the brain, the most important OARs to be integrated were the brain, the brainstem, followed by the eyes. Hypothalamus and pituitary gland were the least important OARs (Supplementary Figure S3).

Regarding the H & N localization, the mandible, parotid glands and spinal cord were the three most important OARs to be integrated, while less than 10% of the participants chose the thyroid gland and the vessels (Supplementary Figure S4). Concerning thoracic irradiation, lungs, heart and spinal cord were the three most important OARs. Noticeably, heart sub-structures were ranked among the three least important OARs with only a 22.2% rate (Supplementary Figure S5).

For abdominal cases, automatic segmentation of the liver, spinal cord and abdominal cavity appeared as the most significant task for participants, closely followed by the kidney. Again, segmentation of the vessels was ranked among the least significant, with a rate of 7.7% (Supplementary Figure S6). Regarding the pelvis localization, the bladder and rectum clearly stood out from other OARs with respective rates of 82.1% and 76.9%, while the first runner-up (sigmoid) was only chosen by 34.2%. The prostate gland for men and the vagina for women were ranked among the five least important OARs (Supplementary Figure S7).

When asked which OARs they would add to currently available structures, the large majority of participants suggested none (88%, 103/117). The anterior interventricular artery and heart sub-structures were the most suggested (3.4%, 4/117).

### 3.4. Definition of Target Volumes

Only 29.9% (35/117) of the participants had an AI solution that proposed the segmentation of target volumes. The large majority of these solutions focused on prophylactic lymph node segmentation (80%), while only 37.1% and 14.3% declared to be proposed with the segmentation of a tumor or invaded lymph node, respectively.

**Figure 4:**
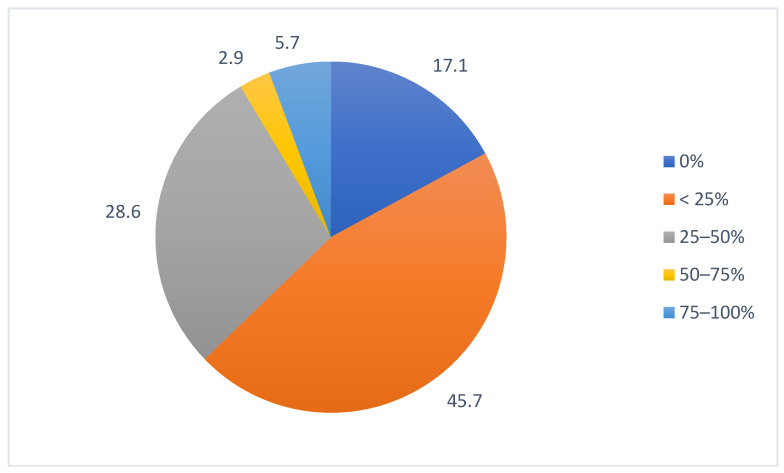
Estimation of time savings regarding target volumes segmentation.

Regarding the segmentation of target volumes, 45.7% of the participants evaluated the time savings to be less than 25%, while 17.1% declared no time gain from AI solutions (Figure 4). When asked about the limitations of AI software for target volumes segmentations, the need for editing (77.1%) and a lack of accuracy (60%) were the two most frequent answers, followed by cost (31.4%) and lack of cross-software integration (20%), as seen in Supplementary Figure S8.

#### 3.4.1. Impact on the Training of Young Radiation Oncologists

Thanks to gains in reproducibility (76.6%) and speed (85.1%), 80.3% of the participants thought that automatic segmentation leads to an improvement in segmentation quality. Only 30.9% of the participants thought that automatic segmentation would improve the accuracy of segmentations.

**Figure 5:**
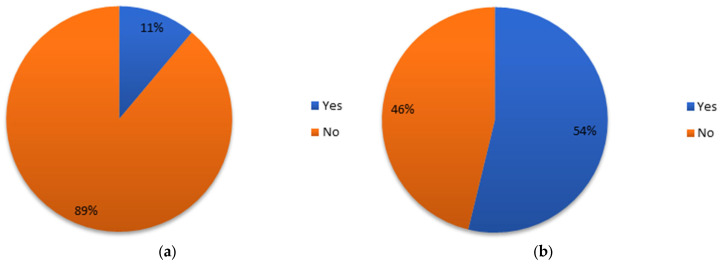
Estimation of automatic segmentation solutions being a menace for radiation oncologists (**a**) and young radiation oncologists training (**b**).

Despite automatic segmentation not being seen as a menace for the marketplace of radiation oncologists for 88.9% of participants, a small majority (53.8%) thought that it could negatively impact the academic training of young radiation oncologists (Figure 5). AI was not integrated into the training of young ROs for 79.5% of them, in contrast to 69.2% and 56.4% of the participants asking for AI tools to be taught directly during ROs training or through university degrees, respectively. Almost a third of participants (30.8%) would limit access to AI solutions during training.

#### 3.4.2. Overall Thoughts of Young Radiation Oncologists on AI Segmentation Solutions

**Figure 6:**
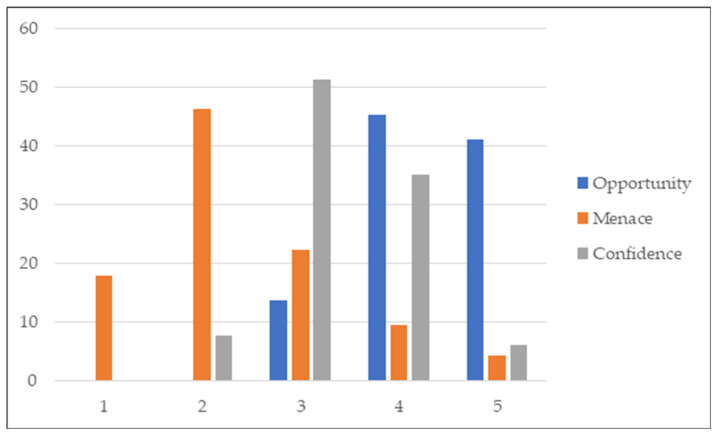
Evaluation of AI segmentation solutions per menace, opportunity and confidence scales.

Young ROs were asked to evaluate AI segmentation solutions based on three different scales: opportunity, menace and confidence. The scales ranged from 1 to 5. For instance, on the opportunity scale, a 1 would mean that the participant felt that the AI solution had no opportunity, while a 5 would mean a very high opportunity. Such solutions were ranked as high to very high opportunities for a large majority of participants (86.3%), with no serious menace for 64.9%. The level of confidence was classified as moderate. (3/5) for 51.3%, while only 6% of the participants had very high confidence in AI-proposed segmentations (Figure 6).

## 4. Discussion

As stated, artificial intelligence profoundly modified the exercise of radiation oncologists. With numerous companies and research teams being involved in this field [16,17,18,19,20], the viewpoint of future clinicians was needed.

With 117 participants representing 35 different and mostly academic centers, this survey is, to our knowledge, the first to focus on young radiation oncologists and their view on AI segmentation tools. Most centers were either already equipped with such solutions or planning to be equipped in the next two years. The growing part of radiation treatment encourages the need for time-saving tools. This survey brings an interesting insight into the views of young ROs but also the challenges that AI tools represent and still face.

AI segmentation software was mostly used for H & N cases, closely followed by breast localizations. Delineation of H & N tumors can be challenging and long for young ROs due to the multiplicity of OAR [21,22,23], explaining the importance of AI segmentation. Caution must be taken as some structures must be delineated using MRI or specific contrast. Breast tumors remain the first cancer treated in radiation service with a high number of patients. Breast and H & N cases were ranked as the most time-saving localizations for approximately 25% of the participants, with a similar number of participants ranking these localizations as the least time-saving. The different levels of performance, according to the tool as well as the users’ expectations, could explain these conflicting results.

Recent dosimetry criteria incite the delineation of more OARs, such as cardiac substructure [24,25,26]. Regular implementation of these “new” OARs might be important in the next years and taken into account for AI segmentation tools. While for the definition of OARs, participants experienced a significant time gain using AI-proposed delineations, with almost 35% of the participants saving between 50–100% of the segmentation time, time gained for target volumes was significantly lower with only 8.6% experiencing such a 50–100% gain. The need for time-saving solutions is high in the field of RT. The efforts should focus on the target volumes’ delineation [18,27]. The next years of RT will be marked by new innovations, such as the broader use of adaptative RT [28] and personalized ultrafractionated stereotactic adaptive radiation therapy (PULSAR) [29]. Brachytherapy could also benefit from automatic delineation before each fraction [30].

Target volumes usually remain more complex, sometimes not well delimitated and with margin depending on anatomic barriers and the natural history of the disease. These characteristics lead to a more difficult automatic realization. Automatic segmentation has two main strengths: speed and reproducibility, but software developers should focus on enhancing the quality. Contours still need to be thoroughly checked and edited, especially target volumes. Concordance with current recommendations must be assessed, as guidelines and consensus can evolve rapidly. Some discordance concerning delineation can be described. These limitations should be known by young ROs.

As suggested by participants, AI segmentation could have a negative impact on the training of young ROs. The majority of them suggested that such tools should be integrated into the training so that future radiation oncologists do not neglect the importance of radioanatomy. Implementation of such tools should follow ESTRO guidelines [31], and centers should share their user experience [32]. AI is not currently implemented in the French oncology radiotherapy cursus. University diplomas or master’s are proposed in parallel. Fully aware of this risk, up to one-third of them even suggested that AI tools should be reserved for senior physicians only, accepting a longer delineation time for the student. On the other hand, automatic segmentation would leave ROs more time for an accurate review of the proposed segmentation, a more precise definition of target volumes and a dosimetric plans assessment.

Several limitations of our survey should be acknowledged. Its length possibly limited the participation rate. Being mandatory for the registration to an H & N dosimetry webinar, answers to the survey are possibly skewed with a selection bias. However, with 117 individual participants, the population represents approximately 50% of the target population. By definition, results were oriented by the propositions made available to the participants. Although it facilitates the quantitative analysis of the data, this may limit the findings.

## 5. Conclusions

We believe this survey on automatic segmentation to be the first to focus on the perception of young radiation oncologists. Software developers should focus on enhancing the quality of proposed segmentations, especially for target volumes, while young radiation oncologists should become more acquainted with these tools and fully apprehend their strengths and weaknesses.

## Figures and Tables

**Table 1:** Submitted Form translated into English.

Question	Choices	
1. I would like to register to the H & N dosimetry webinar	- Yes - No	
2. State your name and surname		
3. State your mail address		
4. State your academic left		
5. State your status	- Resident - Attending - Senior physician	- Foreign Intern - Student - No position
5.1 If Resident a. State your year of training		
5.2 If Attending a. state your year of training	o <2 years o >2 years	
5.3 If senior physician a. State your year of training	o 2–5 years o 5–10 years	o 10–20 years o >20 years
6. In what type of structure do you work?	- University hospital - Academic left specializing in cancer care - General hospital - Private clinic	
7. How many patients per year are treated in your department?	- <800 - 800–1000 - 1000–1500	- 1500–2000 - >2000
8. In your left, who performs the delination of organs at risks (OARs)?	- Dosimetrists and physicists - Mostly residents - Attending or senior physicians mostly - Attending, senior physicians, attendings, physicists and dosimetrists	
9. Is your left equipped with an automatic delination solution?	- Yes - No	
9.1 If your department is equipped with an automatic delineation solution:		
a. How long have artificial intelligence tools for delineation been integrated into clinical routine?	o <1 year o 1–2 years o >2 years	
b. Who has access to these tools in your left?	o All ROs whatever their level of experience o Senior physicians only o Dosimetrists and physicists only o Attending, senior physicians, attendings, physicists and dosimetrists	
c. What is/are the name(s) of the solution available in your department?	o ART-plan Annotate (Therapanacea) o DLCExpert o RayStation automatic segmentation o Mirada Workflow-Box o Elekta ABAS o SPICE	o MIM Maestro o Mvision AI o Limbus AI o Syngo via (Siemens) o Not known
d. What were the motivations for your left to integrate these tools?	o Time saving o Available budget o Improve quality of treatment o Reduce inter-physician variability in contouring o Move towards the international recommendations	
e. For which locations do you use these tools?	o Breast o Prostate o Pelvis o Head and Neck o Thorax	o Neurology o Abdomen o Metastasis o All localizations
f. Rank the following locations in ascending order of time saved (1: little time saved -> 8: maximum time saved)	o Breast o Prostate o Pelvis o Head and Neck	o Thorax o Neurology o Abdomen o Metastasis
g. Using this software saves you time in delineating:	o Macroscopic Tumour volumes (GTV T) o Tumour Target volumes (CTV T) o Macroscopic lymph nodes volumes (GTV N) o Prophylactic lymph nodes volumes (CTV N) o Organs at risk	
h. How much time do you expect to save on OARs delineation (including correction)?	o 0% o <25% o 25–50%	o 50–75% o 75–100%
i. On average, what percentage of the contours should you correct?	o 0% o <25% o 25–50%	o 50–75% o 75–100%
j. Have you ever encountered an error during automatic contouring?	o Yes, laterality error o Yes, aberrant contours (wrong location) o Yes, but only minor and easily correctable errors o No, never	
9.2 If your department isn’t equipped with an automatic delineation solution:		
a. What are the obstacles to the adoption of such a solution?	o Free answer	
b. Have you ever tested/used automatic contouring software?	o Yes o No	
c. How soon do you expect artificial intelligence tools for delineation to be integrated into your left?	o Never o <1 year o 1–2 years o >2 years	
10. Regarding the delineation of organs at risk:		
10.1 Brain: From the following list, what are the 3 organs at risk that must be included in software?	- Brain - Brainstem - Lens - Optic nerve - Eye - Optic chiasma	- Hippocampus - Inner ear - Pituitary gland - Hypothalamus - Lacrymal gland
10.2 H & N: From the following list, what are the 3 organs at risk that must be included in software?	- Temporo-mandibular articulation - Mandible - Oral cavity - Parotid - Larynx - Pharyngeal muscles	- Esophagus - Trachea - Spinal cord - Thyroid - Vessels
10.3 Thorax: From the following list, what are the 3 organs at risk that must be included in software?	- Lungs - Heart - Spinal cord - Esophagus	- Thyroid - Cardiac sub-structures - Vessels
10.4 Abdomen: From the following list, what are the 3 organs at risk that must be included in software?	- Spinal cord - Liver - Pancreas - Spleen - Bowel bag	- Small bowel - Large bowel - Kidneys - Vessels
10.5 Pelvis: From the following list, what are the 3 organs at risk that must be included in software?	- Bladder - Rectum - Sigmoid - Anal canal - Small bowel	- Large bowel - Prostate-Seminal vesicles o vagina-uterus - Lumbosacral Vertebrae-Iliac bones - Vessels
10.6 Which interesting OAR(s) have you never seen in automatic segmentation software?	- Free answer	
11. Regarding the delineation of target volumes		
11.1 Does the software at your disposal offer automatic segmentation of target volumes?	- Yes - No	
11.2 If your software allows the definition of target volumes:		
a. What target volumes are proposed?	o Tumour target volume o Nodal target volume o Prophylactic nodal volume	
b. How much time do you expect to save in contouring the target volumes (including correction)?	o 0% o <25% o 25–50%	o 50–75% o 75–100%
c. What are the limitations of segmentation software?	o Speed o Accuracy o Cost o Lack of cross software integration o Lack of completeness of proposed structures o Need for editing o Learning loss o Other:	
12. Do you think that the use of automatic contouring software improves the quality of the contours?	- Yes - No	
12.1 I think that automatic segmentation can improve the quality of the contours: Why?	- Gain in reproducibility - Gain in speed - Gain in accuracy	
13. Do you think that the use of automatic contouring software is a danger for the profession of radiation oncologists?	- Yes - No	
14. Do you think that the use of automatic contouring software is a danger for the training of young radiation oncologists?	- Yes - No	
15. How do you think automatic delineation tools will impact the training of young radiotherapists?	- Free answer	
16. Did you receive any training on artificial intelligence during your residency?	- Yes - No	
17. In what way (s) do you think the training of radiation oncologists should be adapted?	- Spend less time learning to contour the volumes contoured by the software - Prohibit automatic delineation software at the beginning of the residency - Integrating AI tools into the training - Facilitate personal training on AI (University degree,…) - No changes - Other	
18. Do you think that automatic delineation tools present an additional risk of error in the workflow?	- Yes - No	
19. [Opportunity scale] In your opinion, the integration of artificial intelligence in radiotherapy presents..	- 1 (No opportunity) -> (Very high opportunity)	
20. [Menace scale] In your opinion, the integration of artificial intelligence in radiotherapy presents..	- 1 (No menace) -> (Very high menace)	
21. [Trust scale] In your opinion, the integration of artificial intelligence in radiotherapy presents..	- 1 (No trust) -> (Very high trust)	
22. In a few words, how do you think the radiotherapy specialty will evolve with respect to these artificial intelligence tools?	- Free answer	

## Data Availability

Data available upon request due to restrictions, e.g., privacy or ethical.

## Supplementary Materials

The following are available online at https://www.mdpi.com/article/10.3390/cancers15072040/s1, Supplementary Protocol S1: Form in French with the corresponding English translation; Supplementary Table S1: Checklist for Reporting Results of Internet E-Surveys (CHERRIES); Supplementary Figure S1: Number of new patients per year, on a participant-by-participant analysis; Supplementary Figure S2: Access to automatic segmentation solutions, on a participant-by-participant analysis; Supplementary Figure S3: Ranking of OARs based on their importance—Brain localization; Supplementary Figure S4: Ranking of OARs based on their importance—Head and neck localization; Supplementary Figure S5: Ranking of OARs based on their importance—Thorax localization; Supplementary Figure S6: Ranking of OARs based on their importance—Abdomen localization; Supplementary Figure S7: Ranking of OARs based on their importance—Pelvis localization; Supplementary Figure S8: Limitations of automatic segmentation software regarding target volumes segmentation.
