# Supplementary material for: Excitement and Concerns of Young Radiation Oncologists over Automatic Segmentation: A French Perspective
Source: Cancers (Basel). 2023 Mar 29;15(7):2040. doi: 10.3390/cancers15072040 (PMC10093734; doi:10.3390/cancers15072040)
Supplement: Supplementary file 1 [file cancers-15-02040-s001.zip › cancers-2280688-supplementary.pdf]

SUPPLEMENTARY MATERIALS

Supplementary Protocol S1: Form in French with the corresponding English Translation

Supplementary Table S1: Checklist for Reporting Results of Internet E-Surveys (CHERRIES)

Supplementary Figure S1: Number of new patients per year on a participant-by-participant analysis

Supplementary Figure S2: Access to automatic segmentation solutions on a participant-by-participant analysis

Supplementary Figure S3: Ranking of OARs based on their importance – Brain localization

Supplementary Figure S4: Ranking of OARs based on their importance – Head and neck localization

Supplementary Figure S5: Ranking of OARs based on their importance – Thorax localization

Supplementary Figure S6: Ranking of OARs based on their importance – Abdomen localization

Supplementary Figure S7: Ranking of OARs based on their importance – Pelvis localization

Supplementary Figure S8: Limitations of automatic segmentation softwares regarding target volumes segmentation

Supplementary Protocol S1: Form (original in French, translated into English for publication)

1. Je souhaite m'inscrire au webinaire de Dosimétrie ORL/I would like to register to the H&N dosimetry webinar
   - Oui/Yes
   - Non/No
2. Préciser votre nom et prénom/State your name and surname
3. Préciser votre adresse mail/state your mail adress
4. Préciser votre centre de formation/State your academic center
5. Préciser votre statut/State your status
   - Interne/Resident
   - Chef de clinique ou Assistant/Attending
   - Titulaire/Senior physician
   - FFI/Foreign Intern
   - Elève/Student
   - En disponibilité/No position

5.1 Si Interne/If Resident

   a. Quelle est votre ancienneté ?/State your year of training
      - 1ère année/1st year
      - 2ème année/2nd year
      - 3ème année/3rd year
      - 4ème année/4th year
      - 5ème année/5th year

5.2 Si Chef de clinique ou Assistant/If Attending

   a. Quelle est votre ancienenté ?/state your year of training
      - < 2 ans/< 2 years
      - > 2 ans/> 2 years

5.3 Si titulaire/If senior physician

   a. Quelle est votre ancienneté ?/State your year of training
      - 2-5 ans/2-5 years
      - 5-10 ans/5-10 years
      - 10-20 ans/10-20 years
      - > 20 ans/> 20 years

6. Dans quelle structure travaillez-vous ?/ In what type of structure do you work ?
   - CHU/University hospital
   - CLCC/Academic center specializing in cancer care
   - CHG/General hospital
   - Clinique privée/Private clinic
7. Quel est le nombre de patients traités par an dans votre service ?/ How many patients per year are treated in your department ?
   - < 800

- 800-1000
- 1000-1500
- 1500-2000
- > 2000

8. Dans votre centre, par qui sont contoutés les organes à risque (OARs) ?/In your centre, who performs the delination of organs at risks (OARs) ?
   - Dosimétristes et physiciens/Dosimetrists and physicists
   - Internes le plus souvent/Mostly residents
   - Médecins seniors le plus souvent/Attending or senior physicians mostly
   - Internes, médecins séniors, physiciens et dosimétristes/Attending, senior physicians, attendings, physicists and dosimetrists
9. Votre service est-il équipé d'une solution de contourage automatique ?/Is your centre equipped with an automatic delination solution ?
   - Oui/Yes
   - Non/No

9.1 Si votre service dispose d'une solution de contourage automatique/If your department is equipped with an automatic delineation solution :

a. Depuis combien de temps les outils d'intelligence artificielle pour la délinéation ont été intégrés en routine clinique ?/ How long have artificial intelligence tools for delineation been integrated into clinical routine?
   - < 1an/< 1 year
   - 1-2 ans/1-2 years
   - > 2 ans/> 2 years

b. Qui a accès à ces outils dans votre centre ?/ Who has access to these tools in your centre?
   - Tous les radiothérapeutes quelque soit leur niveau d'expérience/All RadOncs whatever their level of experience
   - Médecins seniors uniquement/Senior physicians only
   - Dosimétristes et physiciens uniquement/Dosimetrists and physicists only
   - Internes, médecins séniors, physiciens et dosimétristes/Attending, senior physicians, attendings, physicists and dosimetrists

c. Quelle/Quelles est/sont le ou les noms de la solution disponible dans votre service ?/ What is/are the name(s) of the solution available in your department?
   - ART-plan Annotate (Therapanacea)
   - DLCExpert
   - RayStation automatic segmentation
   - Mirada Workflow-Box
   - Elekta ABAS
   - SPICE
   - MIM Maestro
   - Mvision AI
   - Limbus AI
   - Syngo via (Siemens)

- o Non connu/Not known

d. Quelles ont été les motivations de votre centre pour intégrer ces outils ?/ What were the motivations for your centre to integrate these tools?
    - o Gagner du temps/Time saving
    - o Budget disponible/Available budget
    - o Améliorer la qualité des plans de traitement/Improve quality of treatment
    - o Diminuer la variabilité des contours entre médecins/Reduce inter-physician variability in contouring
    - o Tendre vers les recommandations internationales/ Move towards the international recommendations

e. Pour quelles localisations utilisez-vous ces outils ?/ For which locations do you use these tools?
    - o Sein/Breast
    - o Prostate/Prostate
    - o Pelvis/Pelvis
    - o ORL/Head and Neck
    - o Thorax/Thorax
    - o Neurologie/Neurology
    - o Abdomen/Abdomen
    - o Métastases/Metastasis
    - o Toutes localisations/All localizations

f. Classer les localisations suivantes par ordre croissant de temps gagné (1: peu de temps gagné -> 8: maximum de temps gagné)/ Rank the following locations in ascending order of time saved (1: little time saved -> 8: maximum time saved)
    - o Sein/Breast
    - o Prostate/Prostate
    - o Pelvis/Pelvis
    - o ORL/Head and Neck
    - o Thorax/Thorax
    - o Neurologie/Neurology
    - o Abdomen/Abdomen
    - o Métastases/Metastasis

g. L'utilisation de ces logiciels vous permet-elle de gagner du temps pour contourer/Using this software saves you time in delineating :
    - o Les volumes tumoraux macroscopiques/Macroscopic Tumour volumes (GTV T)
    - o Les volumes cibles tumoraux/Tumour target volumes (CTV T)
    - o Les volumes ganglionnaires macroscopiques/Macroscopic lymph nodes volumes (GTV N)
    - o Les volumes ganglionnaires prophylactiques/Prophylactic lymph nodes volumes (CTV N)
    - o Les organes à risque/Organs at risk

h. Combien de temps estimez vous gagner pour le contourage des OARs (correction comprise) ?/How much time do you expert to save on OARs delineation (including correction) ?
    - 0%
    - < 25%
    - 25-50%
    - 50-75%
    - 75-100%

i. En moyenne, quel pourcentage des contours devez-vous corriger ?/ On average, what percentage of the contours should you correct?
    - 0%
    - < 25%
    - 25-50%
    - 50-75%
    - 75-100%

j. Avez vous déjà rencontré une erreur lors des contourages automatiques ?/ Have you ever encountered an error during automatic contouring?
    - Oui, erreur de la latéralité/Yes, laterality error
    - Oui, contours aberrants (mauvaise localisation)/Yes, aberrant contours (wrong location)
    - Oui mais uniquement des erreurs minimes et facilement corrigeables/Yes, but only minor and easily correctable errors
    - Non, jamais/No, never

9.2 Si votre service ne dispose pas d'une solution de contourage automatique/If your department isn't equipped with an automatic delineation solution :

a. Quels sont les freins à l'adoption d'une telle solution ?/What are the obstacles to the adoption of such a solution?
    - Réponse libre/Free answer

b. Avez-vous déjà testé/utilisé un logiciel de contourage automatique ?/Have you ever tested/used automatic contouring software?
    - Oui/Yes
    - Non/No

c. Dans combien de temps pensez-vous que des outils d'intelligence artificielle pour la délinéation seront intégrés dans votre centre ?/How soon do you expect artificial intelligence tools for delineation to be integrated into your centre?
    - Jamais/Never
    - < 1 an/< 1 year
    - 1-2 ans/1-2 years
    - > 2 ans/> 2 years

10. Concernant, la définition des organes à risque/Regarding the delineation of organs at risk :

10.1 - Cerveau: Parmi la liste suivante, quels sont les 3 organes à risque à absolument intégrer à un logiciel ?/Brain: From the following list, what are the 3 organs at risk that must be included in software?

- Cerveau/Brain

- Tronc cérébral/Brainstem
- Cristallin/Lens
- Nef optique/Optic nerve
- Œil/Eye
- Chiasma optique/Optic chiasma
- Hippocampe/Hippocampus
- Cochlée/Inner ear
- Hypophyse/Pituitary gland
- Hypothalamus/Hypothalamus
- Glande lacymale/Lacrymal gland

10.2 - ORL: Parmi la liste suivante, quels sont les 3 organes à risque à absolument intégrer à un logiciel ?/H&N: From the following list, what are the 3 organs at risk that must be included in software?

- Articulation temporo-mandibulaire/Temporo-mandibular articulation
- Mandibule/Mandible
- Cavité buccale/Oral cavity
- Parotide/Parotid
- Larynx/Larynx
- Muscles pharyngés/Pharyngeal muscles
- Œsophage/Esophagus
- Trachée/Trachea
- Moelle épinière/Spinal cord
- Thyroide/Thyroid
- Vaisseaux/Vessels

10.3 - Thorax: Parmi la liste suivante, quels sont les 3 organes à risque à absolument intégrer à un logiciel ?/Thorax: From the following list, what are the 3 organs at risk that must be included in software?

- Poumons/Lungs
- Cœur/Heart
- Moelle épinière/Spinal cord
- Œsophage/Esophagus
- Thyroide/Thyroid
- Sous-structures cardiaques/Cardiac sub-structures
- Vaisseaux/Vessels

10.4 - Abdomen: Parmi la liste suivante, quels sont les 3 organes à risque à absolument intégrer à un logiciel ?/Abdomen: From the following list, what are the 3 organs at risk that must be included in software?

- Moelle épinière/Spinal cord
- Foie/Liver
- Pancreas/Pancreas
- Rate/Spleen
- Cavite péritonéale/Bowel bag

- Grêle/Small bowel
- Colon/Large bowel
- Reins/Kidneys
- Vaisseaux/Vessels

10.5 - Pelvis: Parmi la liste suivante, quels sont les 3 organes à risque à absolument intégrer à un logiciel ?/Pelvis: From the following list, what are the 3 organs at risk that must be included in software?

- Vessie/Bladder
- Rectum/Rectum
- Sigmoide/Sigmoid
- Canal anal/Anal canal
- Grêle/Small bowel
- Colon/Large bowel
- Prostate-Vésicules séminales ou vagin-utérus/Prostate-Seminal vesicles o vagina-uterus
- Vertèbres lombo-sacrées-Ailes iliaques/Lombosacral vertebrae-Iliac bones
- Vaisseaux/Vessels

10.6 Quel(s) OAR(s) vous paraissant intéressant n'avez-vous jamais vu dans un logiciel de segmentation automatique ?/ Which interesting OAR(s) have you never seen in automatic segmentation software?

- Réponse libre/Free answer

11. Concernant, la définition des volumes cibles/Regarding the delineation of target volumes

11.1 Le logiciel à votre disposition propose-t-il la segmentation automatique des volumes cibles ?/Does the software at your disposal offer automatic segmentation of target volumes?

- Oui/Yes
- Non/No

11.2 Si votre logiciel permet la définition des volumes cibles/If your software allows the definition of target volumes :

a. Quels volumes cibles sont proposés ?/What target volumes are proposed?
    - Volume tumoral/Tumour target volume
    - Volume ganglionnaire macroscopique/Nodal target volume
    - Volume ganglionnaire prophylactique/Prophylactic nodal volume
b. Combien de temps estimez-vous gagner pour le contourage des volumes cibles (correction comprise) ?/How much time do you expect to save in contouring the target volumes (including correction)?
    - 0%
    - < 25%
    - 25-50%
    - 50-75%
    - 75-100%

c. Quelles sont les limites des logiciels de segmentation ?/ What are the limitations of segmentation software?
    - Rapidité/Speed
    - Précision/Accuracy
    - Cout/Cost
    - Manque d'intégration inter-logiciel/Lack of cross software integration
    - Manque d'exhaustivité des structures proposées/Lack of completeness of proposed structures
    - Nécessité de correction/Need for editing
    - Perte d apprentissage/Learning loss
    - Autre/Other :

12. Pensez-vous que l'utilisation de logiciel de contourage automatique permet d'améliorer la qualité des contours ?Do you think that the use of automatic contouring software improves the quality of the contours?
    - Oui/Yes
    - Non/No

12.1 Je pense que la segmentation automatique peut améliorer la qualité des contours/ I think that automatic segmentation can improve the quality of the contours : Pourquoi/Why ?
    - Gain en reproductibilité/Gain in reproducibility
    - Gain en rapidité/Gain in speed
    - Gain en précision/Gain in accuracy

13. Pensez-vous que l'utilisation de logiciel de contourage automatique est un danger pour le métier d'oncologues-radiothérapeutes ?/ Do you think that the use of automatic contouring software is a danger for the profession of radiation oncologists?
    - Oui/Yes
    - Non/No

14. Pensez-vous que l'utilisation de logiciel de contourage automatique est un danger pour la formation des jeunes oncologues-radiothérapeutes ?/Do you think that the use of automatic contouring software is a danger for the training of young radiation oncologists?
    - Oui/Yes
    - Non/No

15. Selon vous, comment les outils de délinéation automatiques risquent d'impacter la formation des jeunes radiothérapeutes ?/How do you think automatic delineation tools will impact the training of young radiotherapists?
    - Réponse libre/Free answer

16. Avez-vous bénéficié d'une formation sur l'intelligence artificielle au cours de votre internat ?/ Did you receive any training on artificial intelligence during your residency?
    - Oui/Yes
    - Non/No

17. De quelle(s) façon(s) pensez-vous qu'il faudrait adapter la formation des radiothérapeutes ?/ In what way(s) do you think the training of radiation oncologists should be adapted?
    - Passer moins de temps à apprendre à contourer les volumes contourés par le logiciel/Spend less time learning to contour the volumes contoured by the software

- Interdire les logiciels de délinéation automatique en début de cursus/ Prohibit automatic delineation software at the beginning of the residency
- Intégrer les outils d'IA dans la formation/Integrating AI tools into the training
- Faciliter les formations personnelles sur l'IA (DU/DIU/M2)/ Facilitate personal training on AI (University degree,…)
- Aucune adaptation/No changes
- Autre/Other :

18. Pensez-vous que les outils de délinéation automatique présentent un risque d'erreur supplémentaire dans le work-flow ?/ Do you think that automatic delineation tools present an additional risk of error in the workflow?
    - Oui/Yes
    - Non/No
19. [Echelle d'opportunité] Selon vous, l'intégration de l'intelligence artificielle en radiothérapie présente...
    [Opportunity scale] In your opinion, the integration of artificial intelligence in radiotherapy presents..
    - 1 (Aucune opportunité/No opportunity) -> (Très forte opportunité/Very high opportunity)

20. [Echelle de menace] Selon vous, l'intégration de l'intelligence artificielle en radiothérapie présente...
    [Menace scale] In your opinion, the integration of artificial intelligence in radiotherapy presents..
    - 1 (Aucune menace/No menace) -> (Très forte menace /Very high menace)

21. [Echelle de confiance] Selon vous, l'intégration de l'intelligence artificielle en radiothérapie présente...
    [Trust scale] In your opinion, the integration of artificial intelligence in radiotherapy presents..
    - 1 (Aucune confiance/No trust) -> (Très forte confiance /Very high trust)

22. En quelques mots, comment pensez-vous que la spécialité de radiothérapie évoluera vis-à-vis de ces outils d'intelligence artificielle ?/ In a few words, how do you think the radiotherapy specialty will evolve with respect to these artificial intelligence tools ?
    - Réponse libre/Free answer

Supplementary Table S1. Checklist for Reporting Results of Internet E-Surveys (CHERRIES).

Design	Describe survey design	Done
Institutional Review Board (IRB) approval and Informed consent process	IRB approval	Not required
	Informed consent	Information was provided on the first page of the survey
	Data protection	No personal information was stored
Development and pre-testing	Development and testing	Methodology for the development of the survey is detailed in the manuscript.
Recruitment process and description of the sample having access to the questionnaire	Open survey versus closed survey	Closed survey
	Contact mode	E-mail
	Advertising the survey	E-mail: mandatory for the registration to a webinar that was advertised using e-mails and other communication platforms (Twitter, Facebook, web page,….)
Survey administration	Web/E-mail	Web
	Context	Mandatory for registration to a webinar that was advertised using e-mails and other communication platforms (Twitter, Facebook, web page,….)
	Mandatory/voluntary	Mandatory
	Incentives	Mandatory for registration to a webinar
	Time/Date	Registrations to the webinar (and thus access to the survey) were opened two months before the webinar took place
	Randomization of items or questionnaires	Not feasible
	Adaptive questioning	Yes
	Number of Items	20 with sub-items
	Number of screens (pages)	4-5, depending on the answers
	Completeness check	All items had a mandatory answer
	Review step	Respondents were able to review their answers
Response rates	Unique site visitor	No participation rates are provided
	View rate (Ratio of unique survey visitors/unique site visitors)	
	Participation rate (Ratio of unique visitors who agreed to participate/unique first survey page visitors)	
	Completion rate (Ratio of users who finished the survey/users who agreed to participate)	
Preventing multiple entries from the same individual	Cookies used	No
	IP check	No
	Log file analysis	No
	Registration	Yes
Analysis	Handling of incomplete questionnaires	Not used
	Questionnaires submitted with an atypical timestamp	Not applicable
	Statistical correction	Not used

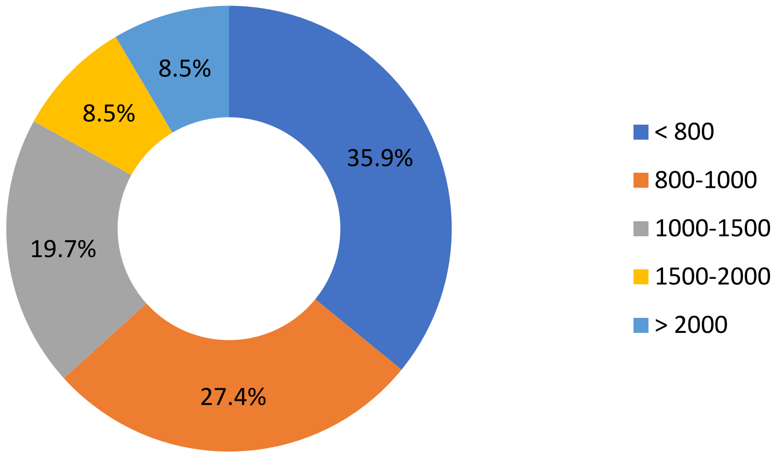

Supplementary Figure S1. Number of new patients per year on a participant-by-participant analysis.

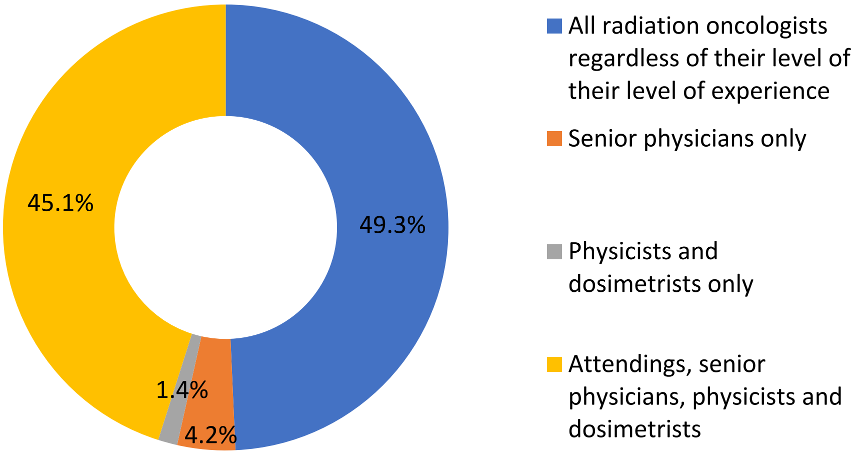

Supplementary Figure S2. Access to automatic segmentation solutions on a participant-by-participant analysis.

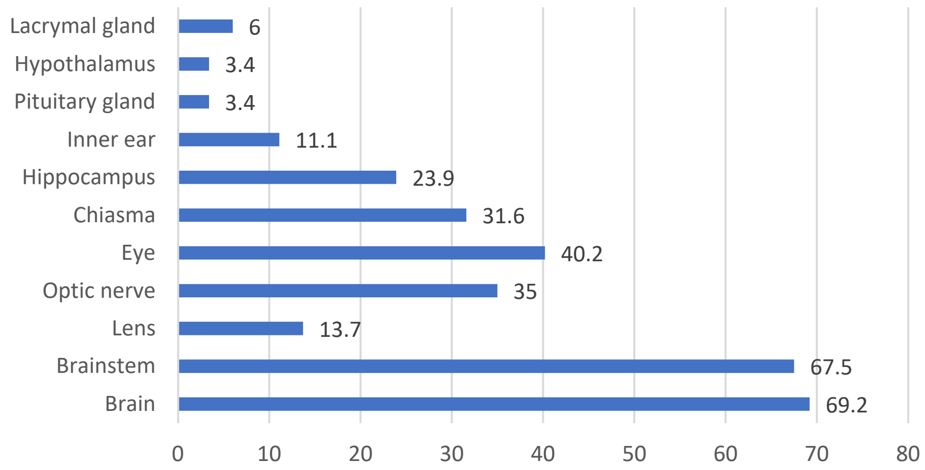

Supplementary Figure S3. Ranking of OARs based on their importance – Brain localization.

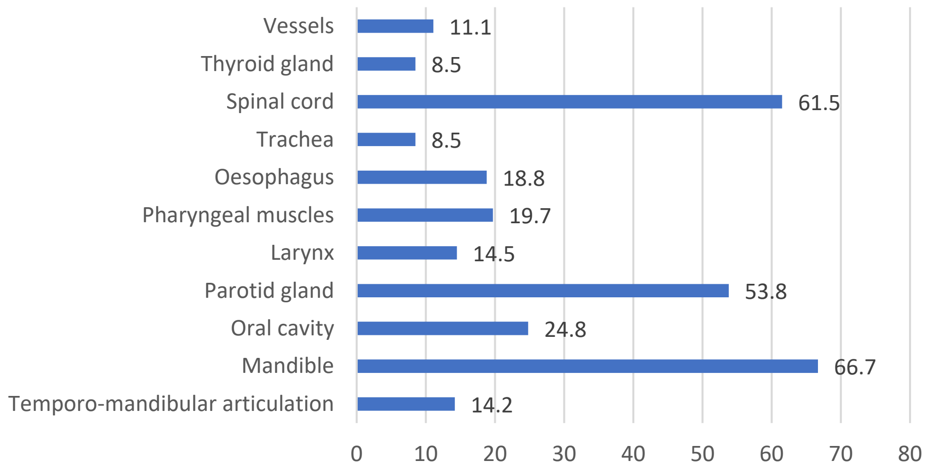

Supplementary Figure S4. Ranking of OARs based on their importance – Head and neck localization.

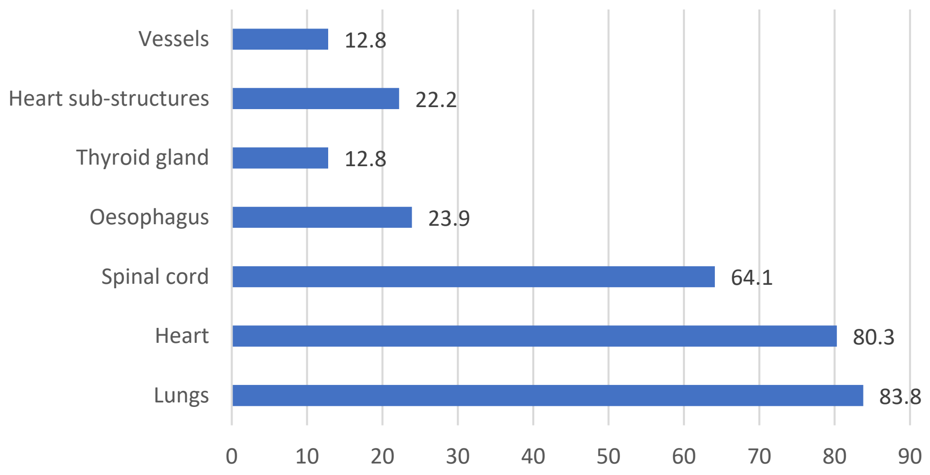

Supplementary Figure S5. Ranking of OARs based on their importance – Thorax localization.

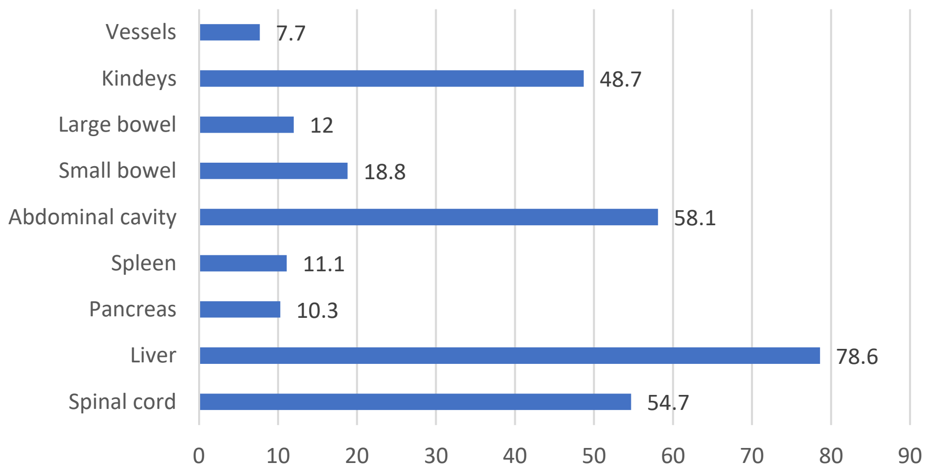

Supplementary Figure S6. Ranking of OARs based on their importance – Abdomen localization.

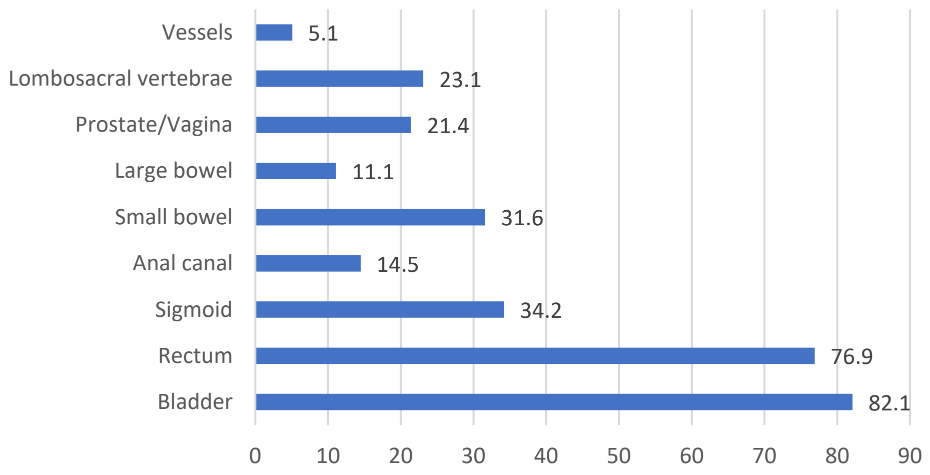

Supplementary Figure S7. Ranking of OARs based on their importance – Pelvis localization.

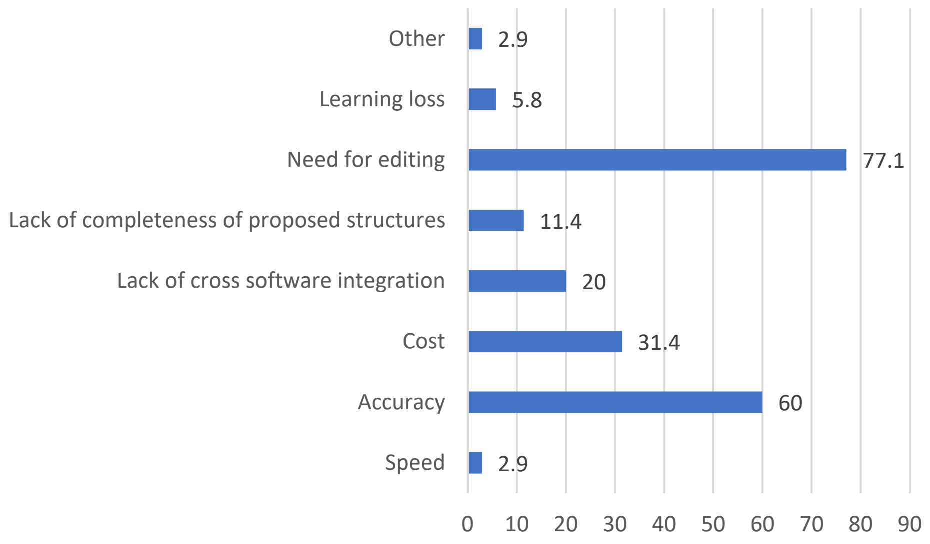

Supplementary Figure S8. Limitations of automatic segmentation software regarding target volumes segmentation.
